# Performance Optimization Approach of Polymer Modified Asphalt Mixtures with PET and PE Wastes: A Safety Study for Utilizing Eco-Friendly Circular Economy-Based SDGs Concepts

**DOI:** 10.3390/polym14122493

**Published:** 2022-06-19

**Authors:** Faizan Mushtaq, Zhian Huang, Syyed Adnan Raheel Shah, Yinghua Zhang, Yukun Gao, Marc Azab, Sajid Hussain, Muhammad Kashif Anwar

**Affiliations:** 1 State Key Laboratory of High-Efficient Mining and Safety of Metal Mines, University of Science and Technology Beijing, Ministry of Education, Beijing 100083, China; s20201530@xs.ustb.edu.cn (F.M.); zhangyinghuaustb@sina.com (Y.Z.); gaoyukunustb@sina.com (Y.G.); 2 Department of Civil Engineering, Pakistan Institute of Engineering and Technology, Multan 66000, Pakistan; kashifanwar723@gmail.com; 3 College of Engineering and Technology, American University of the Middle East, Egaila 54200, Kuwait; marc.azab@aum.edu.kw; 4 College of Transportation Engineering, Tongji University, Shanghai 201804, China; sajidbhatti1214@gmail.com

**Keywords:** sustainability, polymer wastes, polyethylene terephthalate (PET), composites, mechanical properties, SDGs, eco-friendly

## Abstract

Eco-friendly waste utilization helps in the development of sustainable infrastructures. Recently, researchers have focused on the production of road infrastructures using the circular economy concept of human safety. The objective of this study is to investigate and explore the utilization of optimum polymer waste content for the development of polymer-modified asphalt mixtures using response surface methodology (RSM). RSM based on Box–Behnken design (BBD) was employed to optimize experimental design and included three factors: X1, polymer type; X2, polymer contents; and X3, testing day. The optimized responses determined by the RSM were as follows: MS of 42.98 kN, MF of 5.08 mm, and MQ of 8.66 kN/mm, indicating a favorable and consistent precision in comparison with experimental values. Moreover, the Marshall characteristics of samples prepared with PE were quite improved compared to PET. In conclusion, the incorporation of such polymer wastes in road construction is a sustainable and cost-effective way of improving their engineering properties. This study will help in the development of sustainable road infrastructures supporting human safety and environmentally friendly practices.

## 1. Introduction

Bitumen, also known as asphalt binder, is a petroleum by-product produced during the distillation of hydrocarbon chains. It has valuable properties, such as long-term durability, high stability, and water resistance, which allow it to be used as a road building material [1]. Bitumen is used with aggregates as a binding material in the production of asphalt mixtures for infrastructure projects. The performance parameters and ultimate stability of asphalt pavements are heavily reliant on the binder’s functionality. The degradation of the bitumen binder causes a wide range of road pavement failures such as cracking failures, rutting, and fatigue cracking [1,2]. Moreover, the increasing cost of bitumen has significant effects on the overall project cost.

**Figure 1:**
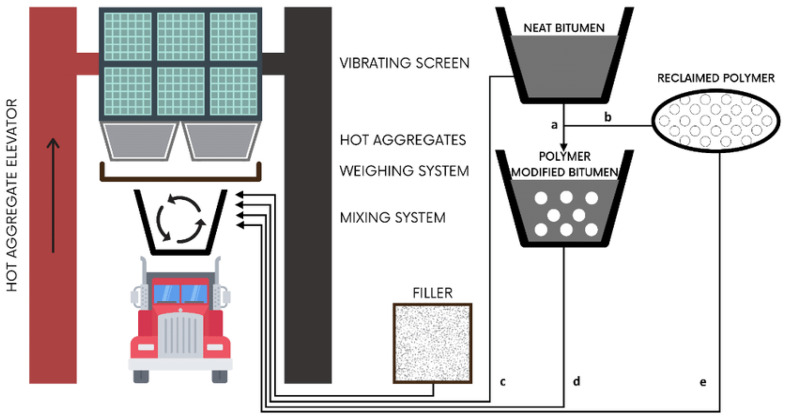
Polymer modification process of mixing by both wet and dry method [24].

Although the repairs and maintenance cost of road pavement are unfavorable for socioeconomic and climatic reasons, much effort is expended to minimize such aforementioned failures. A significant number of researchers have studied bitumen alteration in order to achieve improved durability and high stability and strength. In the past, countless initiatives have been explored, and scholars have effectively utilized numerous waste materials e.g., crumb rubber [3,4], nylon and polymer waste [5,6,7], polyethylene [8,9], polypropelene [10], recycled PET [11,12], polystyrene [13], and ordinary plastic bottles [14,15,16], etc. The positive enhancement of all these leading bituminous qualities that are crucial for road construction is recognized as a primary goal of all these investigations [17,18]. Polymer matrices of bitumen are one of the most suitable and widely used approaches among all reported modification approaches [19]. To produce polymer-based asphalt, the polymer is mixed into the bituminous mix by a chemical process (wet method) or mechanical mixing (dry process) as shown in Figure 1 [20,21]. The polymer and asphalt are directly incorporated at elevated temperatures for a specific duration of time in the wet procedure to ensure adequate chemical or physical reactions between ingredients. The wet process integrates plastic in the shape of flakes, pellets, or powder into warm bitumen when a polymer is processed from plastic wastes [22]. Plastics chosen for the wet method often require melting ranges just below the defined range, owing to standard blending temperatures between 160 to 170 °C [12,23].

Response surface methodology (RSM) is becoming a common practice in the construction sector as a more effective way of assessing and optimizing outcome measures [25]. Tan et al. [26] employed central composite design, also known as the CCD approach, in combination with the RSM model to find the best raw ingredient ratio for several asphalt mixture test parameters. The impacts of the prepared factors, such as grading, aggregation, and other factors, on the indirect tensile strength (ITS) of hot mix bituminous compositions using RSM were researched and assessed by Kavussi et al. [27]. Hamzah et al. [28,29,30] used the CCD of RSM to explore the effects of recovered aggregate ratio, compaction temperature, and bitumen percentage on the physical and mechanical parameters of asphalt pavement. In addition, Hamzah et al. [29] discovered that statistical methods built on RSM can accurately predict the impacts of aging on binder viscoelastic performance at extreme temperatures. RSM was also used and studied by another researcher [31] to assess the results of lime concentration and gradient on the ITS and its ratios in an asphalt mix under dry and wet situations. RSM-based statistical model was used to optimize the binder concentration and gradation of asphalt mixture using the tensile strength rate. RSM can be performed to effectively analyze bituminous binder and combination based on comprehensive literature [32].

The statistical method based on RSM to quantify the optimum polymer contents of PET and PE wastes in the pavement mixture is still not presented in the literature. Therefore, the main objective of this study is to optimize the polymer-modified asphalt mixture made with PET and PE wastes by employing the statistical approach of RSM. This statistical technique can measure the relationships and correlations among several input parameters by taking into consideration the contributions of two or more variables in an investigation. The results are examined and investigated theoretically, experimentally, and hypothetically using a statistical approach based on RSM to give a better understanding of these additives and polymer contents on Marshall test parameters. Moreover, this research aims to meet the eco-friendly, efficient, and economical requiremens for the development of road networks that will be helpful for all countries to utilize their plastic waste as a binder (additive) for the construction of extremely durable, inexpensive road systems, as well as reducing air and noise pollution in metropolitan areas.

## 2. Background

### 2.1. Literature Review

The flexible pavement comprises multiple materials, including bitumen (binder), filler, and fine and coarse aggregates. For the effective production of asphalt concrete mix, the percentage of each material appears to be most significant for efficient and economical construction objectives [33]. It is important to ascertain the fundamental properties of every ingredient for a better understanding and development of the asphalt mix. The load-bearing material of asphalt concrete is recognized as an aggregate because around 95% of aggregate by weight is involved in the production of asphalt mix [34]. In civil engineering projects, aggregates are generally utilized for almost every type of work, including concrete production, asphalt mix, railway ballast, road formation, etc.; they are in-active chemical materials that are blended with various binding substances to form concrete, asphalt mix, mortar, etc. [35,36,37].

Typically, each type of road deteriorates over time, principally due to countless considerations including severe weather situations, traffic loading, and asphalt and bitumen quality [38,39]. Worldwide, numerous scholars have investigated countless modifiers to enhance the quality and performance of bitumen through the utilization of a variety of modified bitumen blends. The literature shows a few studies that utilized additives such as fatty acid amides, sulfur, polyphosphoric acid, etc. [40,41,42,43]. Meanwhile, other polymers a utilized to enhance the characteristics of bitumen; the most efficient and widely utilized polymers for modification are recognized as styrene–butadiene rubber (SBR), styrene–butadiene–styrene copolymer (SBS), etc. [44,45,46,47]. For many experimental and theoretical analyses, polyethylene and ethylene–vinyl acetate have also been utilized [48]. The practical properties of normal bitumen have been enriched through the application of these valuable modifiers. Additionally, the costs of bitumen processing and road construction have been affected by the blending of additives with bitumen. The foremost aim of the entire process should be the evaluation of economic benefits [49]. Currently, the global enduring target of sustainability can be achieved through the application of waste raw materials or additives to systematically handle inadequate natural resources [50]. The aggregate is a key part of asphalt pavements obtained from natural resources. A large investigation was performed to study the utilization of recycled and solid waste materials as an alternative to aggregates through an assortment of raw materials such as glass, finely crushed waste bricks (FCWB), steel waste slag (SWS), crumbed rubber tires (CRT), etc. [45,51,52,53]. As mentioned above, several polymers successfully used to enrich the basic bitumen characteristics at an advanced level, such as polypropylene and polystyrene through useful reclaimed geo-membranes, have also been employed to stimulate anticipated attributes in bitumen [52,54,55,56,57]. The general properties of bitumen also improve through the accumulation of natural rubber [58]. Concerning the utilization of recycled materials, the recycled waste polyurethane foam has been efficiently employed as a substitute for bitumen in asphalt pavements; in addition, its durability has been examined for lightweight mortars [59,60,61,62,63].

In asphalt pavement, because of weak adhesion between bitumen and aggregates, the deterioration and destruction of road pavement surfaces occurs. After the advancement of adhesion, the phenomenon of stripping, concerning the surface tension among aggregate and binder, seems to be improved, reducing the surface deterioration. In the past, through statistical approaches, many asphalt pavement distress attributes, such as rutting, cracking, and shoving, etc. have been investigated as the cause of failure in these pavements [64,65]. The key cause of these distress attributes is the selection of inadequate mixing proportions, which leads to the deterioration of these pavements; otherwise, the best service quality of these pavements can be achieved through correct ingredient proportions [66,67]. In the case of hot mix asphalt pavement, the temperature variations have precisely impacted the viscosity of bitumen and then the adhesion properties are altered through this variation, which disturbs the bond between aggregates and bitumen [68]. The low stability of asphalt concrete leads to several types of distress, and the stability of specimen is identified through the maximum load resistance that can be borne by specimen before the failure [65]. Scientifically, the Marshall-based stability and tensile strength of specimens are related to each other, because the testing arrangement of the Marshall and the indirect tensile test appears to be similar, although the sample rested in the Marshall test head lies within a specified limit [69,70,71]. Conversely, countless constraints, such as the shape and size of the aggregates, weather variations, grade and content of bitumen, as well as loading situation of traffic, etc., also influence the stability of asphalt pavements. Through the application of stability tests, the rapid and efficient evaluation of the elastic behavior of asphalt concrete layers can be achieved [72]. Worldwide, this approach can be extensively utilized, although there are overwhelming constraints, and it is an expensive and time-consuming method [73,74,75].

The use of statistical methods based on Response Surface Methodology (RSM) to quantify the optimum polymer contents of PET and PE wastes in the pavement mixture is still not found in the literature. Therefore, the main objective of this study is to optimize the polymer-modified asphalt mixture made with PET and PE wastes by employing the statistical approach of RSM.

### 2.2. Application of Circular Economy Concepts and Sustainable Development Goals

Sustainable Development Goals, also known as SDGs, are the roadmap toward achieving greater global sustainability by 2030 [76]. SDGs are based on 17 global goals that are directly interlinked with 169 benchmarks. These goals are progressively being used as a foundation for organizing and promoting sustainability practices by both private and state sector stakeholders around the world. Similarly, the concept of the Circular Economy (CE) has recently become more popular to achieve sustainability at local and international levels [12,77]. Several drivers, such as states, municipalities, and many multinational organizations, are actively studying and promoting ways to practice CE initiatives on the global scale [78].

Few research papers were found when we searched for details about the connection between the CE and the SDGs [79,80]. The study examines the extent to which CE initiatives are significant for achieving the SDGs in developing countries based on an in-depth literature overview. The study concluded that CE concepts can be used as a toolbox and concrete action tactics for reaching a significant number of SDGs. The immediate relationship or collaborations among CE and SDGs are found in SDG 6: clean water and sterilization, SDG 7: affordable and clean energy, SDG 8: respectable work and financial development, SDG 12: sustainable utilization and creation, and SDG 15: life on the planet [79]. These five SDGs are explained one by one as follows:

- The treatment of wastewater is a promising approach to the CE model based on the SDG 6 to address water pollution and increase current water supply–demand through recycling and re-use in the future.
- SDG 7 is to ensure that everyone has access to affordable, efficient, renewable, and smart energy.
- SDG 8 promotes sustainable economic growth and decent work for everyone.
- SDG 12 is to ensure a sustainable framework of consumption as well as the production process.
- SDG 15 is relevant to the restoration of natural capital and terrestrial ecosystems in a sustainable way.

**Figure 2:**
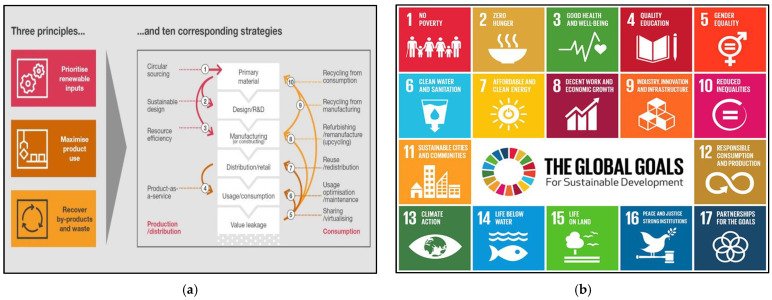
(**a**) Three principles with ten corresponding strategies regarding circular economy [81]. (**b**) SDGs [76].

The four other SDGs indirectly linked to the CE model are SDG 1 (no poverty), SDG 2 (zero hunger), SDG 11 (sustainable cities and communities), and SDG 14 (life below water). The production phase of circularity is based on four approaches (1 to 4) that attempt to maximize the usage of renewables while minimizing value loss across the life cycle. The consumption phases (such as sharing, re-using, repairing, re-manufacturing, and recycling) of circularity are based on six approaches (5 to 10) that prevent value leakage by rotating products and materials at their optimum utility levels. The three basic principles and ten corresponding strategies regarding circular economy [81] and SDGs are presented in Figure 2.

## 3. Materials and Methods

**Figure 3:**
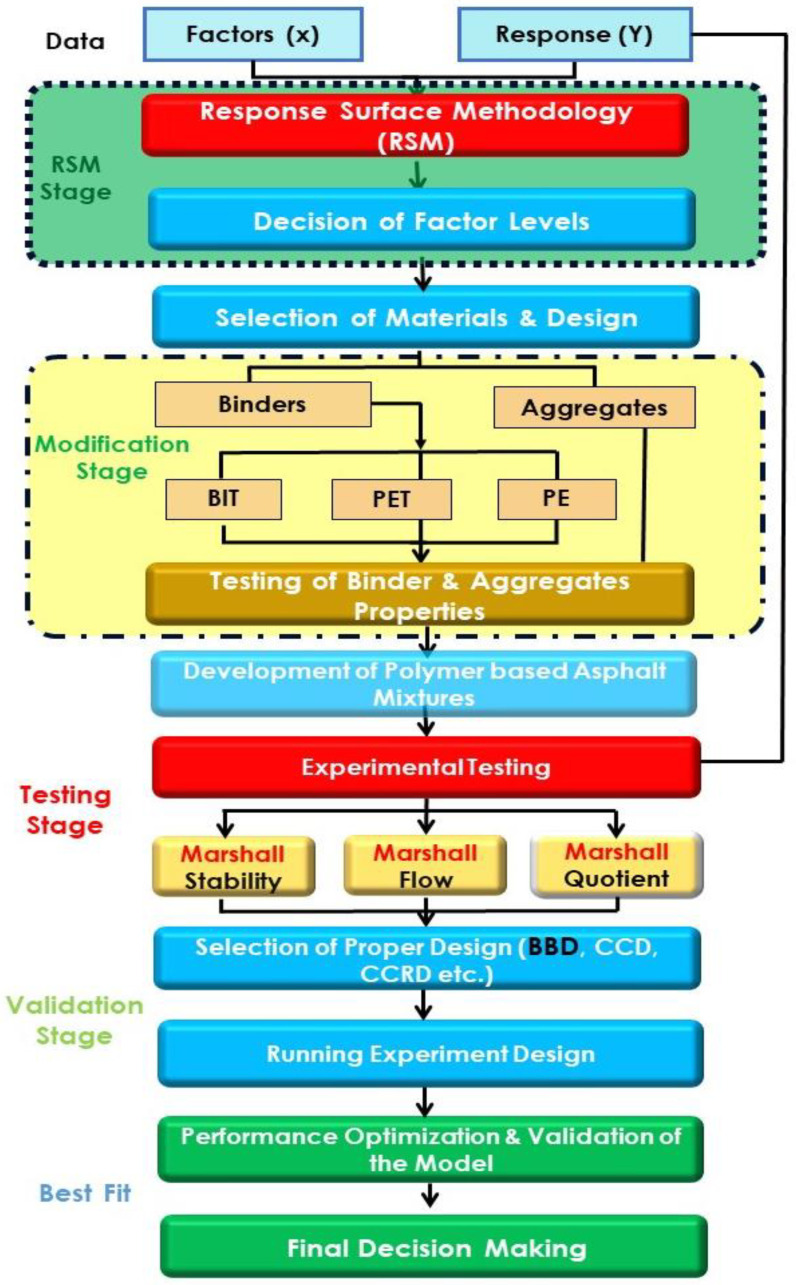
Flow chart of research methodology.

In this study, the research methodology has been divided and performed into two stages: polymer-modified bitumen and polymer-modified asphalt mixes. The evaluation of various plastic contents is performed by both fresh and mechanical testing. The engineering properties, such as Marshall stability flow and stiffness of polymer-modified asphalt mixtures, were comprehensively examined for the in-depth analysis of their behavior and performance under local environmental conditions. The research methodology of the current study is presented stepwise in the form of the flowchart in Figure 3.

### 3.1. Basic Materials

Bitumen is a sticky and highly viscous petroleum liquid, black in color, and taken from deep natural deposits as well as acquired through the fractional distillation of crude oil while boiling at 525 °C. It is composed of hydrocarbon compounds. Worldwide, around 70% of bitumen is utilized for asphalt pavements as a binder; the remaining 30% is utilized for many applications, including building protection, waterproofing, etc. [82,83]. In this investigation, 60/70 grade bitumen was used as a binder, and an optimal percentage content of 7% binder was found. For a detailed understanding of binder behavior, its physical properties were examined through the laboratory equipment according to the ASTM standard listed in Table 1 [84,85,86,87,88].

Sakhi Sarwar aggregate, which comprises 55% limestone and 45% sandstone, has been utilized as a coarse aggregate in this study. The coarse aggregate size varied from 20–4.75 mm, and the maximum size of fine aggregate or grains was <4.75 mm, which was from Chenab, Punjab. The detailed aggregate properties identified according to the ASTM standards are listed in Table 1 [88,89,90,91,92,93,94].

### 3.2. Bitumen Modification

The present study considers two waste materials for the modification of bitumen: polyethylene terephthalate (PET) and polyethylene (PE). The first additive, PET, was developed through the shredding of waste plastic beverage bottles into small pieces that are often found in garbage or water or even taken from waste dealers. The next additive, PE, was developed through the shredding of flexible plastic pipes locally available at an economical cost. Normally, after the long-term use of pipes, they become waste and people will dump them in garbage. Shredding these additives can transform them into a usable form [72]. The percentage substitution of bitumen with each additive ranged from 5–20% at each testing period. At each proportion of additive, bitumen, and additive were thoroughly mixed at a temperature of 160–170 °C.

### 3.3. Testing and Preparation of Specimens

**Figure 4:**
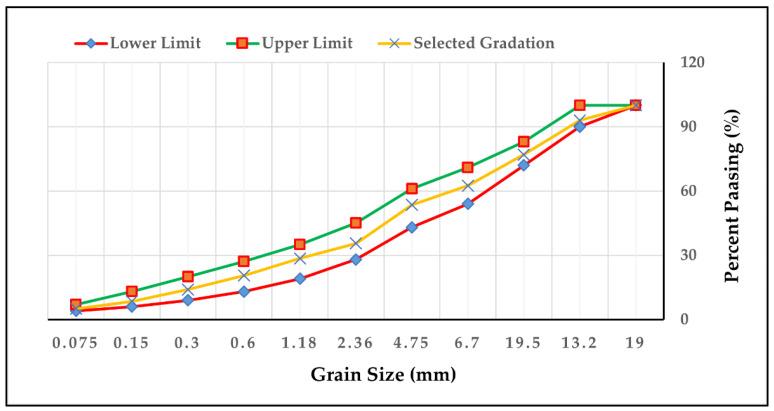
Aggregates gradation curve.

For the Marshall stability analysis, the specimen was prepared in the transportation laboratory through the help of an asphalt mixer and the casting of modified and unmodified bitumen specimens was performed simultaneously. For the homogenous mixing of ingredients of the Marshall cake, the aggregates were first dried in an oven at a constant temperature of around 150–170 °C; then, for the removal of air voids, the bitumen was heated at a constant temperature of 160–170 °C. All these heating processes were performed according to the ASTM standards [56,95]. For one Marshall cake, approximately 1200 g of aggregate was taken from the aggregate blend, and the characteristics of the binder and aggregates utilized for the asphalt mix preparation are listed in Table 2. Through the asphalt mixer apparatus, the temperature variation during the mixing of ingredients has been managed and controlled. In this study, 60/70 grade bitumen was used and the mixing of asphalt ingredients was performed at an approximate temperature of 165 °C. After homogenization and the preparation of asphalt mix, the mix was laid into preheated metal molds, and mechanical compaction was performed at 100–140 °C, around 75 blows were applied on both sides of the specimen by using a standard compactor hammer for impact loading. Later, when the specimen cooled, it could be extracted from the mold with the help of a hydraulic extractor available in the transportation laboratory [95]. For testing purposes, the sample was submerged into a thermostatically controlled water bath at a constant temperature of 60 °C, for 30–40 min preceding the testing. In this study, at each percentage substitution of bitumen with additives, the specimens were tested over two periods, the minimum and maximum testing periods were 1 day and 30 days, respectively. The Marshall testing of all specimens at each period was performed according to the ASTM standard D-1559 for precise determination of stability and flow value [72,96]. The aggregate gradation curve is presented in Figure 4. Further, three samples were prepared for each blend of asphalt mix in each testing period, and the average of these was taken as the final value. Various statistical approaches were additionally employed to explore the performance of the produced asphalt mix [97,98,99,100,101,102].

### 3.4. Marshall Characteristics

The standard testing procedure was adopted for measuring the Marshall characteristics such as Marshall stability (MS) as the major variable, Marshall flow (MF) to help determine the testing performance, and the Marshall quotient (MQ) of both modified asphalt mixtures using the Marshall stability machine. The MQ is the ratio of stability to flow and is measured in kN/mm as an indicator of resistance to the cracking and deformation of asphalt mixtures.

### 3.5. Response Surface Methodology

**Figure 5:**
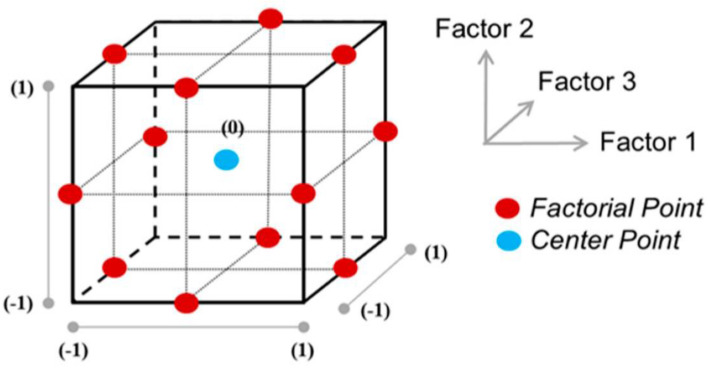
The layout of Box–Behnken design with three factors [103].

Response surface methodology (RSM) is a statistical approach for boosting random research design, and it is used to explore the quantitative link among factors (independent) and response variables. Box–Behnken design (BBD) was utilized on the experimental asphalt mixtures dataset to find the optimum percentage of both polymer content and asphalt mix design. By introducing center points into the plan or system, the BBD creates a hybrid of two randomized block design strategies. The center runs, also known as NC, is a trial base in which the central point is (0, 0, …, 0), and has no less than three NCs with different sums of the k-factor. If three factors are used, the BBD design equals 12 + a center run as shown in matrix form below in Equation (1). A three-variable layout for the BBD is shown in Figure 5.

(1) $$D=\left[\begin{array}{ccc} -1 & -1 & 0\\ -1 & 1 & 0\\ 1 & -1 & 0\\ 1 & 1 & 0\\ -1 & 0 & -1\\ -1 & 0 & 1\\ 1 & 0 & -1\\ 1 & 0 & 1\\ 0 & -1 & -1\\ 0 & -1 & 1\\ 0 & 1 & -1\\ 0 & 1 & 1\\ 0 & 0 & 0 \end{array}\right]$$

The experimental design in this study used the Box–Behnken approach to optimize the experimental dataset using RSM. The RSM was applied on 24 experimental runs. Each factor and the quadratic model vary considerably on three levels. The regression analysis and response surface graph were plotted using Design Expert Software (13 versions, Stat-Ease Inc., Minneapolis, MI, USA). In the second step, we identified the major effect and relationship among each variable on the response using the second-order quadratic polynomial equation as presented below (Equation (2)).

(2) $$Y=\beta_{0}+\sum_{i=1}^{n}\beta_{i}X_{i}+\sum_{i<j}^{n}\beta_{ij}X_{i}X_{j}+\sum_{j=1}^{n}\beta_{jj}X_{j}^{2}$$

Here, “*Y*” is the response variable; *X_i_* and *X_j_* are the independent variables; the mean response coefficient is represented by $\beta_{0}$; the number of factors is called “*n*”; and $\beta_{ij}$ is the linear relationship between independent variables. In this study, three factors were studied as independent variables: *X*_1_, Polymer Type; *X*_2_, Polymer content %; and *X*_3_, Day on which the Marshall stability was performed. The Marshall characteristics (such as Marshall stability, Marshall flow, and Marshall stiffness) are the response variables.

## 4. Results and Discussion

### 4.1. Performance Analysis of Polymer-Modified Bitumen

**Figure 6:**
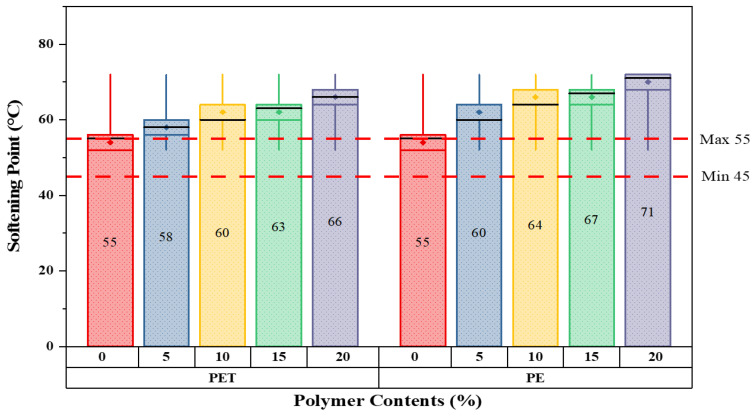
Softening point values with varying plastic content percentages.

**Figure 7:**
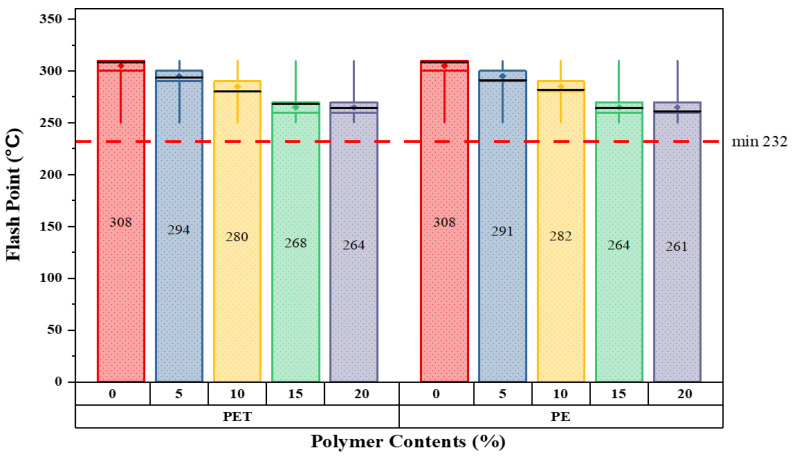
Flashpoint values with varying plastic content percentages.

**Figure 8:**
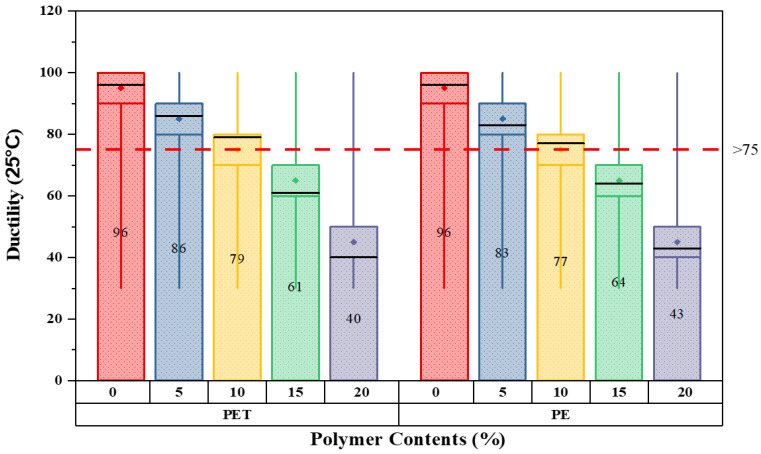
Ductility values with varying plastic content percentages.

**Figure 9:**
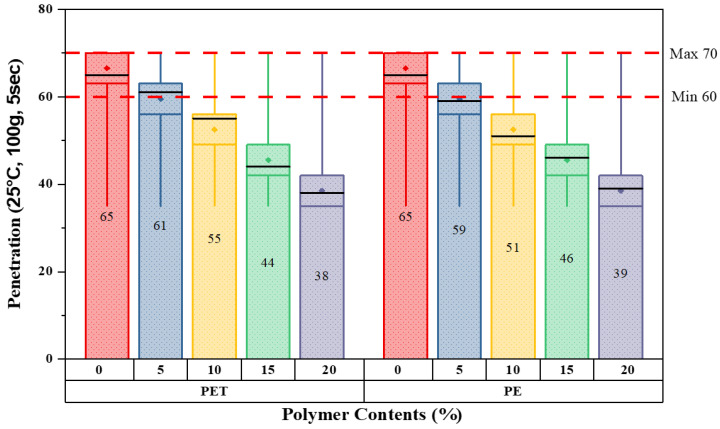
Penetration values with varying plastic content percentages.

The primary step is to investigate the characteristics of modified bitumen according to ASTM standards. All the basic modified bitumen properties are examined through the help of laboratory equipment; the outcomes are presented in Figure 6, Figure 7, Figure 8 and Figure 9.

The key aim of this in-depth examination is to understand the behavior of modified bitumen at each percentage substitution. Because of the elastic–plastic nature of bitumen, basic testing is considered to be essential in this study to examine the alteration of binder (bitumen) at each proportion in both waste materials. The results demonstrate that as the percentage substitution of both modifiers increases, the softening point increases simultaneously. In contrast, the flash and fire point values lie within the standard ASTM range. At 10% substitution for either materials, the ductility values satisfy the standard limits; afterwards, the outcomes are below the specified limits. Hence, the analysis concludes that the values of waste material-modified bitumen lie close to the standard range specified by ASTM. It should be remembered that a higher melting point of bitumen is occasionally better in hot climate situations because it resists bitumen bleeding.

Figure 6 shows the softening point readings for five different matrix forms of modified bitumen as measured in °C. Overall, the readings of softening points increase with increased percentages. The softening point values at 0% are the lowest, around about 55, and are within the standard range, which ranges between 45–55 for 60/70 grade bitumen. Moreover, a significant rise in softening point values can be seen for the addition of both plastics. This value then rose sharply to reach a minimum of just about 23% at 20% replacement of PE waste content as compared to neat bitumen. A similar trend was observed in the case of PET-modified bitumen.

The above graph illustrates the flashpoint readings for five various matrix forms of modified bitumen as calculated in unit °C (Figure 7). Overall, the flashpoint values decreased as the percentage increased. The standard value for flashpoint is 232 °C. The flashpoint values at 0% were maximum for the control samples. Moreover, a dramatic fall in flashpoint values can be seen with addition of both plastics. This value then fell steadily to reach a minimum of just about 16% for 20% replacement of PE waste content as compared to neat bitumen. Similarly, a similar line pattern was seen in the case of PET-modified bitumen with values quite close to PE at a similar percentage level.

Figure 8 illustrates the ductility results for five various matrix types of modified bitumen as determined in units of cm @ 25 °C. Overall, the results of ductility fell for both plastic wastes over the percentages given. The standard value for ductility at 25 °C in cm is >75 cm. The ductility values at 0% were maximum for the pure binder with a penetration grade of 60–70. Additionally, a dramatic drop in ductility results can be seen for the case of both waste plastic modifiers. This value then fell steadily to reach a minimum of 59% for 20% replacement of PET waste content as compared to neat bitumen. An analogous pattern was found for PE-modified bitumen.

Figure 9 illustrates the penetration results for five various matrix types of modified bitumen as determined in units of °C. Overall, the results of penetration decreased for both plastic wastes as the percentages increased. The standard value for softening point at 25 °C is between 60–70. The penetration values at 0% were maximum for the pure binder of grades 60–70. Moreover, a dramatic fall in penetration results can be seen in the case of both plastic wastes addition. This value then fell steadily to reach a minimum of just about 42% at 20% replacement of PET waste contents as compared to the pure bitumen binder. Likewise, a comparable line pattern was observed for PE-modified bitumen, but the values were lower than PET before falling to 15%. This then increased to a peak of 1 degree higher than for PET.

### 4.2. Analysis and Optimization of Polymer Modified Bitumen Using RSM

The RSM was employed to explore the effects of polymer-modified asphalt mixtures under local climatic conditions on the Marshall stability indices, such as Marshall stability (MS), Marshall flow (MF), and Marshall quotient (MQ). By using Box–Behnken design (BBD), 24 experimental runs were designed and the overall series (runs) of experimental sets was considerably reduced. The experimental design and the Marshall characteristics of polymer-modified asphalt mixtures obtained from the experimental testing are described in Table 3. Three factors—PT: Polymer types i.e., PET (0) and PE (1), PC: Polymer contents (0–20%), and Testing days (1 and 30)—were used as an independent factors, and MS (kN), MF (mm), and MQ (kN/mm) were the response variables used in this study.

### 4.3. Statistical Analysis and ANOVA Results

The best-fitted regression model was recommended and adopted for additional analysis based on the above study design and experimental results using BBD. It was selected based on the R-square value, *p*-value, and F-value, between multiple response variables including linear, quadratic, 2FI, and cubic polynomial equations. In this study, the suggested model is 2FI which is used to perform the ANOVA analysis for each independent factor and their effect or interaction on the mechanical characteristics of polymer-modified asphalt mixtures measured in terms of response variables. The level statistical of significance was set as 0.05, meaning that models and factors are significant if the *p*-value is less than 0.05. In this research, *p*-values less than 0.05 were considered to be significant. The quadratic model appears to be the best, according to the RSM design, and these variables are significant. The ANOVA results are presented in Table 4.

### 4.4. Marshall Stability Analysis of Modified Bitumen

The ANOVA for the response variable MS (kN) is described in Table 4. The findings show that the 2FI model of MS has acceptable fitting values, such as a *p*-value < 0.0001. The model F-value of 605.04 implies the model is critical. A *p*-value less than 0.0500 indicates model terms are critical. In this case, A, B, C, AB, AC, and BC are significant variable model terms. Values above 0.1000 demonstrate that the model terms are not significant. The predicted R^2^ of 0.9895 agrees with the adjusted R^2^ of 0.9942; for example, one factor that matters is an adequate precision of 0.2, indicating the signal to noise proportion. A proportion more than 4 is attractive. Our ratio of 63.197 indicates an adequate signal. The regression coefficients of variables can be calculated using the least-squares approach. The appropriate Equation (3) concerning actual variables for MS (kN) is then determined by leaving out the irrelevant variables as follows:

(3) Y1:+4.72+0.6476A+0.0417B+0.5811C+0.6476AB+0.0706AC−0.149BC

**Figure 10:**
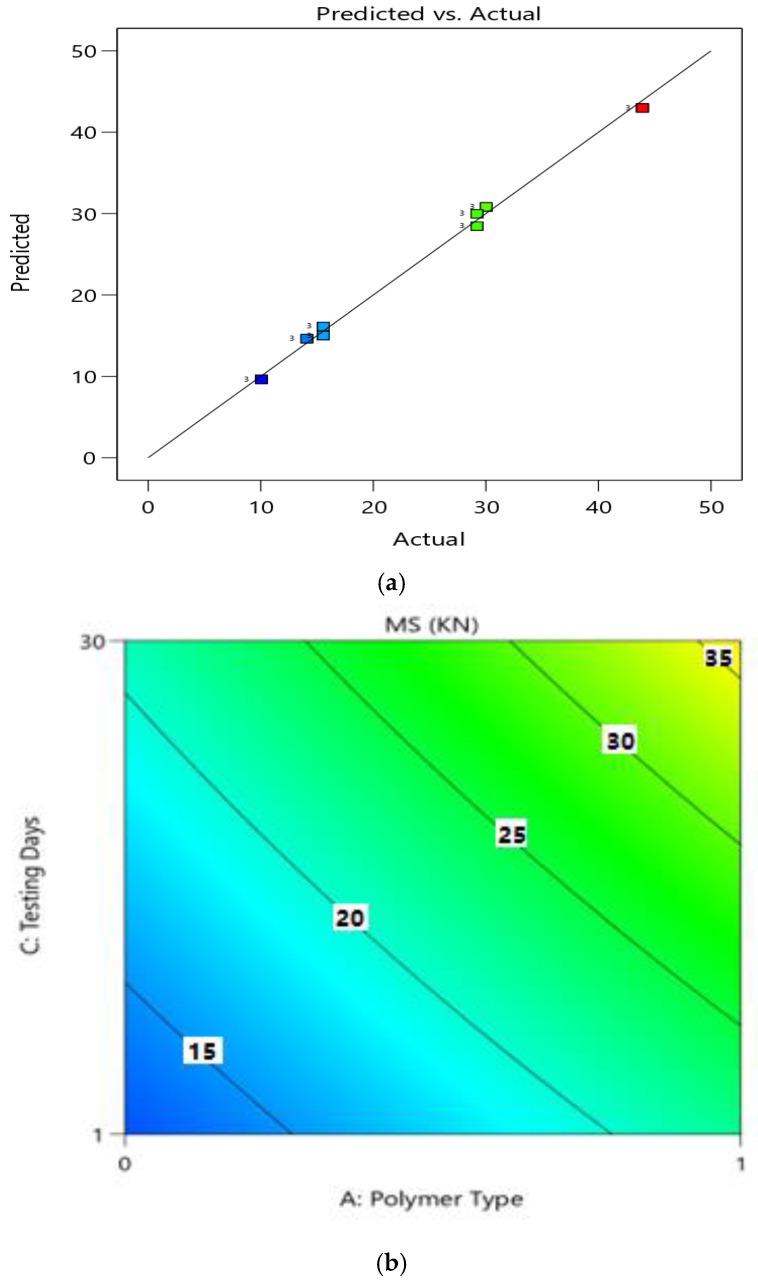
Predicted vs. actual plot (**a**) for MS (kN); (**b**) Contour plot (2D) between the MS (kN) and Factors.

**Figure 11:**
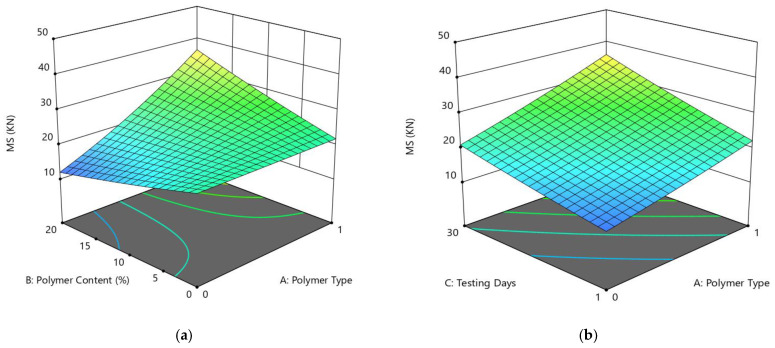

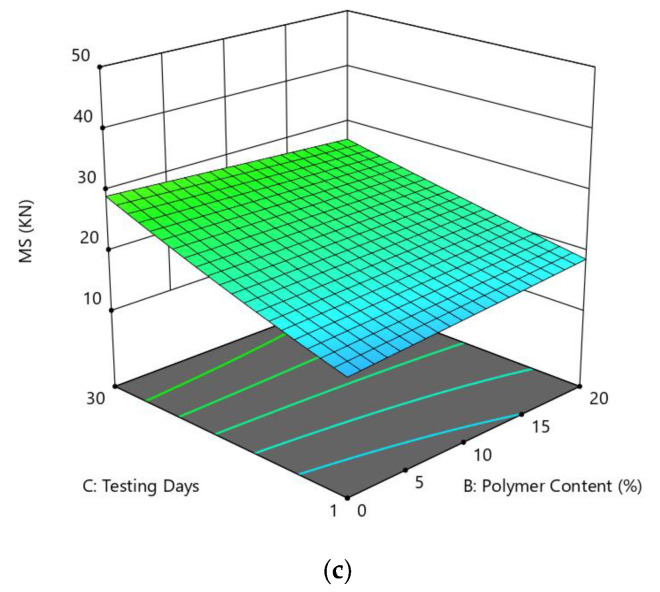
3D response surface plots for the MS (kN) vs. (**a**) AB, (**b**) AC, (**c**) BC factors.

Figure 10a shows the diagnostics of the predictive model, which shows an almost linear distribution of the data, suggesting a strong significance. From the ANOVA, the variables of polymer type (A), polymer content (B), and day (C) were significant in the model results i.e., *p*-value < 0.05, and the term polymer type had the most significant impact on Marshall stability, with a *p*-value of <0.0001 (which is also less than 0.05). Using the RSM approach, the response surface plots (2D and 3D) of MS are presented in Figure 10b and Figure 11 to explore relationships between the multiple factors and response variables (MS). The correlation among preparation variables and MS, and the interaction of setup factors, are displayed using the 2FI fitting model. Figure 10a shows that the stability of modified asphalt mixtures is increased with an increase in polymer content from 0 to 20% in the case of both polymer materials. The results for PE-modified materials were better as compared to PE wastes. The interactions of factors A (polymer type) and B (testing day) on the Marshall stability as shown in the 3D graph (Figure 10b) reveal an increase in values with the change in polymer type and testing days (i.e., after 30 days). Figure 10 shows the 3D plot between the interactions variables B (polymer content) and C (day), confirming improved MS with the change in both polymer contents after 1 and 30 days of testing.

### 4.5. Marshall Flow Analysis of Modified Bitumen

The ANOVA for the response variable MF (mm) is detailed in the second row of Table 4. The model F-value of 33,359.91 implies the model is significant. A *p*-value less than 0.0500 demonstrates model terms are significant. In this case, A, B, C, AB, AC, and BC are significant variable model terms. Values above 0.1000 indicate that the model terms are not significant. The predicted R^2^ of 0.9998 agrees with the adjusted R^2^ of 0.9999; for example, one factor that matters is an adequate precision of 0.2, indicating the signal to noise proportion. A proportion more than 4 is attractive (desirable). Our ratio of 412.739 indicates an adequate signal. The significant considerations in the model equation of MF, namely X1, X2, X3, X1X3, X1X2, and X2X3, were determined using the *p*-values. The appropriate Equation (4) concerning the actual variables for MS (kN) is then determined by leaving out the irrelevant variables as follows:

(4) Y2:+2.08−0.011−0.223B+0.3916−0.0109AB−0.0039AC−0.0314BC

**Figure 12:**
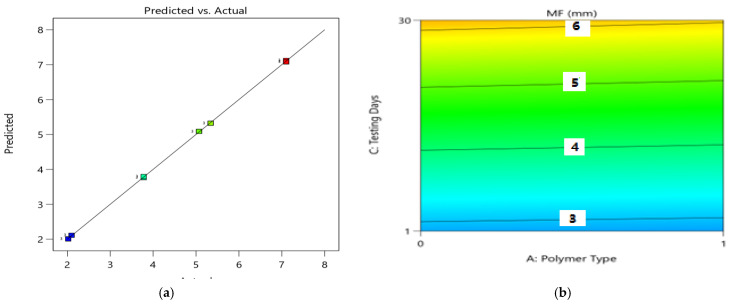
(**a**) Predicted vs. actual plot for MF (kN); (**b**) contour plot (2D) between the MF (mm) and three factors.

**Figure 13:**
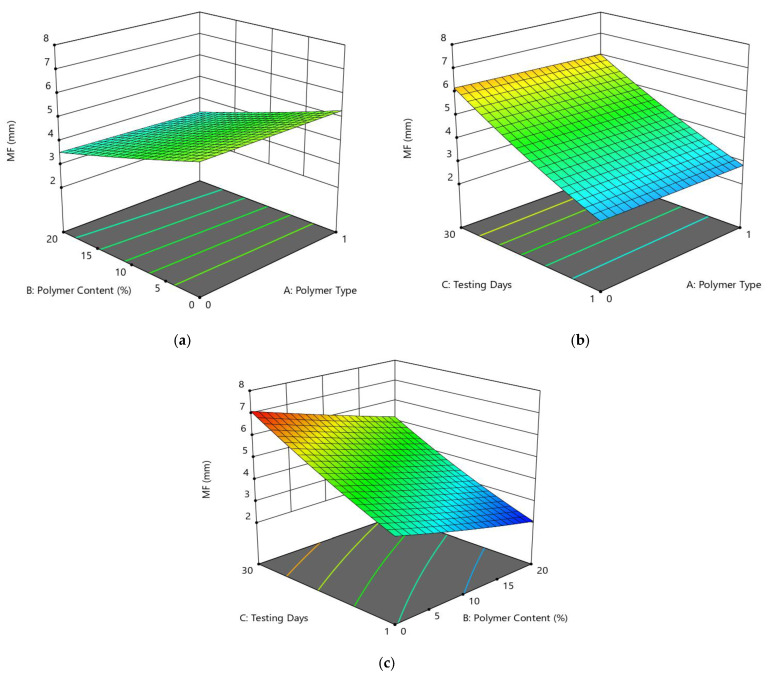
3D response surface plots for the MF (mm) vs. (**a**) AB, (**b**) AC, (**c**) BC factors.

The condition with coded variables can be utilized to make forecasts about the reaction for given levels of each component. The coded condition is valuable for recognizing the general effect of the elements by contrasting the variable coefficients. Figure 12a presents the diagnostics of the predictive model, which shows a close dispersion of datasets, suggesting a good significance relation. Based on ANOVA results, the linear 2FI variables of polymer type (A), polymer content (B), and testing day (C) showed significant model results, i.e., *p*-value > 0.05, and these variables had strong significant effects on Marshall flow values with *p*-values of <0.05. Using the RSM approach, the response surface plots (2D and 3D) of MF responses are presented in Figure 12b and Figure 13 to explore relationships between the multiple variables and Y-response (MF). The correlation among design factors and MF (mm) and the interaction of setup factors are displayed using the fitting 2FI model. Figure 13a shows that flow values of modified asphalt mixtures are increased with an increase in polymer content from 0 to 20% in the case of both polymer materials. The results for PE materials showed stiffer properties compared to PE wastes. Therefore, PE-modified binders and asphalt mixtures showed better resistance to deformation.

After the detailed experimental work, the average outcomes of Marshall stability and flow values for both additive specimens after 1-day and 30-day periods are presented in Figure 11, Figure 12 and Figure 13. The quadratic model of MF has nonsignificant fitting values; the *p*-value is 0.4435, which is greater than 0.05. In brief, Figure 11, Figure 12 and Figure 13 show the variations in stability and flow values for each testing period scenario of each additive. In the 1-day testing scenario, for replacement by PET, the stability and flow values are increased at 5% replacement and then decreased, but acceptable according to ASTM standard. Comparatively, in the case of PE, the stability values increase; in fact, at 20% proportion there is an approximate 93% increase in stability, compared to the unmodified specimen, and then the flow value decreased. Conversely, in the 30-day testing scenario for PE, the stability values increase by around 47–93% in contrast to 1-day testing consequences, but all are satisfactory according to the ASTM standard, although the flow values are outside of the range. The same trend can be seen for the 30-day testing scenario for PET.

### 4.6. Marshall Quotient Analysis of Modified Bitumen

The Marshall quotient (MQ) is defined as the ratio of Marshall stability (kN) to flow (mm) and is used to determine rutting resistance. The ANOVA results of the Marshall quotient are presented in the third row of Table 4. The statistical results show that the quadratic model of MQ has acceptable fitting values, such as a *p*-value less than 0.05. The model performance based on the F-value of 849,978,602,645.14 suggests the model is significant. There is just a 0.01% chance that a F-value this enormous could happen because of clamor (noise). A *p*-value under 0.0500 indicates that model terms are significant. For this situation, A, B, C, AB, AC, BC, and ABC are significant variable model terms. Values more than 0.1000 indicate that the model terms are not significant. The significant considerations in the model equation of MQ, namely X1, X2, X3, X1X3, X1X2, and X2X3, were established using the *p*-values. The appropriate Equation (5) with actual variables for MQ (kN/mm) is then determined by exclusion of the irrelevant variables as follows:

(5) Y3:=+5.93+2.01A+1.81B−1.05C+2.01AB−0.506AC−1.04BC−0.507ABC

**Figure 14:**
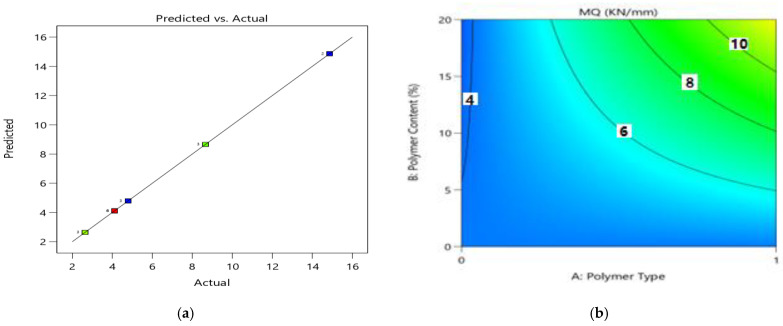
(**a**) Predicted vs. actual plot for MQ (kN/mm) (**b**) contour plot (2D) between the MQ (kN/mm) and three factors.

**Figure 15:**
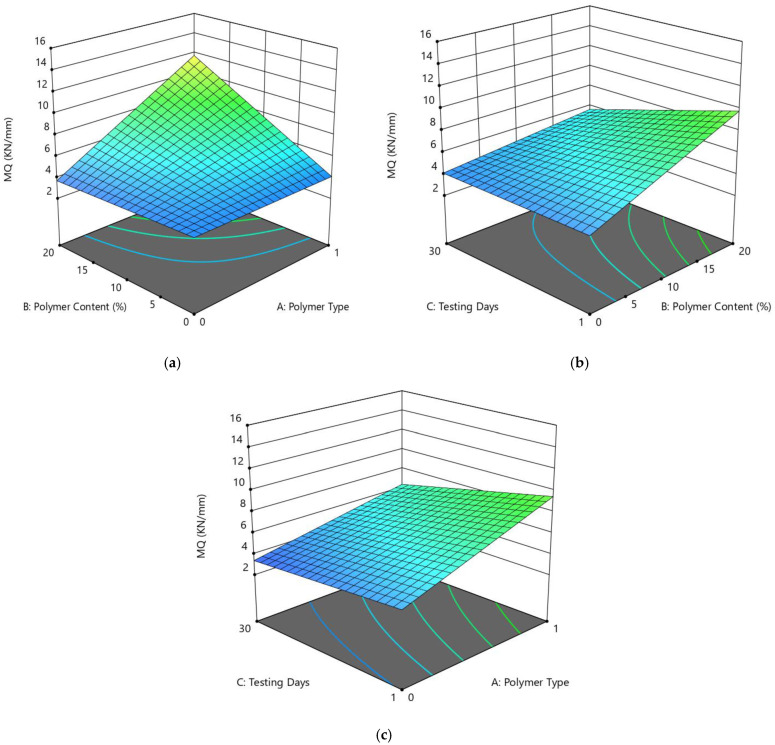
3D-Response surface plots for the MQ (kN/mm) and three factors (**a**) AB, (**b**) AC, (**c**) BC.

The relationships for coded elements can be utilized to make forecasts about the reaction for given levels of each component. Of course, the elevated degrees of the variables are coded as +1 and the low levels are coded as −1. The coded condition is valuable for distinguishing the general effect of the elements by looking at the variable coefficients. Figure 14a shows the diagnostics of the predictive model, which shows an almost linear distribution of the data, suggesting a strong significance. As per the ANOVA model, the variables of polymer type (A), polymer content (B), and day (C) showed significant model results, i.e., *p*-value < 0.05, and the linear term of all variables also has a significant impact on MQ with a *p*-value of <0.05. Using the RSM approach, the response surface plots (2D and 3D) of the MQ variable are presented in Figure 14b and Figure 15 to explore the relationships between the multiple variables and MQ. The correlation among design factors and MQ (kN/mm) and the interaction of setup factors are displayed using the fitting quadratic regression model.

The effects of factors A (polymer type), B (polymer content), and C (testing days) on the Marshall quotient (MQ) is shown on the 3D graph (Figure 14). The experimental results showed that MQ values increased with an increase in waste plastic contents up to 20% of PE addition, as shown in the contour plot of Figure 14a. The results show that the waste polymer improves the Marshall stability of the asphalt mixtures, but also has a considerable detrimental impact on flow values, consistent with earlier findings. Based on experimental results, the increased MS and MQ of the polymer-modified asphalt could be associated with greater PET and PE dispersion in the asphalt through shear blending, which could provide higher stiffness and contribute to improved stability. The increased MQ values showed more stiffness characteristics and more resistance against deformation. The Marshall characteristics are thought to be primary measures of road performance, as better road performance is attributed to greater stability and reduced flow. We can conclude that the asphalt compositions treated with plastic wastes will have superior performance attributes based on the above findings.

### 4.7. Optimization of Polymer-Modified Asphalt Mixtures of PET and PE

**Figure 16:**
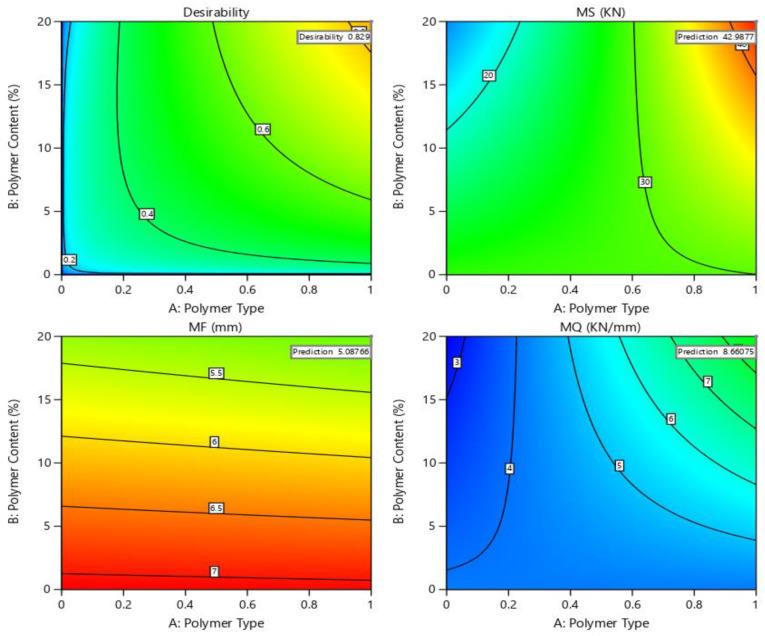
Optimization values of responses vs. multiple variables using RSM.

Regarding the objective of the study, Figure 16 demonstrates the outcomes of optimizing polymer content (PET and PE), as much as is feasible, in Marshall properties. As previously stated, the prepared factors have diverse effects on the Y-variables for both polymer-modified asphalt mixtures and the respective influence significant levels are also diverse. A multi-response optimization approach was performed based on the best-suited 3D-RSM to establish the optimum combination of preparatory factors. The predicted and experimental values for multiple factors concerning corresponding responses are presented in Table 5. The BBD-based RSM can then be used to determine the best blend of prepared setups. The following was identified as the best blend of preparatory factors based on the laboratory settings.

### 4.8. Environmental Impact Analysis of Polymer-Modified Bitumen

The heat transfer of road pavements gives constant dynamical changes with periodic environmental conditions because of exposure to the actual environment. The heat is conducted via the base course, road foundation, and sub-grade during this change, which ultimately results in a dynamic and complex temperature profile. Hence, the temperature aspect of the road surface design should be considered as a temperature distribution zone under outside weather changes. As far as isotropic granular materials are concerned, the uneven heat flow in 3-D space obeys the conservation law of energy and Equation (6) of Fourier partial differential. In other words, the time-dependent heat phenomena of road pavement are defined by the differential equation given below.

(6) $$\rho \cdot c\frac{\partial T}{\partial t}-\lambda \cdot (\frac{\partial^{2}T}{\partial x^{2}}+\frac{\partial^{2}T}{\partial y^{2}}+\frac{\partial^{2}T}{\partial z^{2}})-Q=0$$

Here, *ρ* = Density of asphalt material in unit kg/m^3^.

*c* = Specific heat constant of asphalt material in unit J·kg^−1^·k^−1^.

*λ* = Thermal conductivity in-unit W·m^−1^·K^−1^.

*x*, *y*, *z* = Three-dimensional coordinates.

*Q* = 0, which is the heat produced per unit volume.

In simultaneous analysis with the equation of the conduction of heat, the heat transfer of the pavement surface can be determined by the differential of Equation (1). The initial conditions of thermodynamics of the asphalt can generally be divided into two forms depending on the thermodynamic resistivity theory. When the temperature of the road surface is defined to be the boundary, the thermodynamic criterion can be considered to be the first form, such as the Dirichlet condition, and described as:

(7) $$T\left(x,y,z,t\right)=\bar{T}\left(t\right)$$

Here, $\bar{T}$ is the assigned temperature of the pavement surface at °C.

After this heat flux volume at the demarcation line of the road pavement has been assigned, the thermodynamic flow pattern can be considered in a second form, such as the Neumann condition, and can be represented as:

(8) $$\lambda \left(\frac{\partial T}{\partial x}n_{x}+\frac{\partial T}{\partial y}n_{y}+\frac{\partial T}{\partial z}n_{z}\right)=\bar{q}\left(t\right)$$

**Figure 17:**
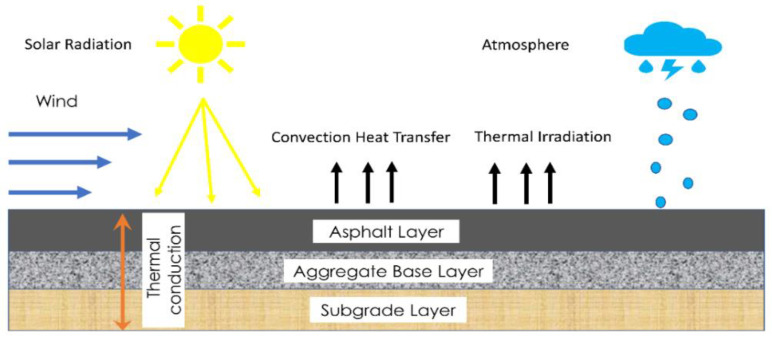
Effect of temperature variations on road surface due to climatic conditions.

Here, $n_{x},n_{y},\;\mathrm{and}\;n_{z}$ are cosines directional, which are the normal outer to the road surfaces respectively and $\bar{q}$ stands for a density of heat flux in W/m. As for the road surface subjected to the open environment, *q*(*t*) on the surface of the road pavement comprises solar energy ($q_{x}$), convection heat transfer ($q_{c}$) and thermal irradiation ($q_{r}$) as shown in Figure 17 and these can be calculated from the following equation.

(9) $$q_{s}=\{\begin{array}{l} 0,0\leq t<12-c/2\\ q_{0}cos\;m\omega \left(t-12\right),12-c/2\leq t\leq 12+\\ 0,12+c/2\leq t\leq 24 \end{array}c/2$$

Here, $q_{0}$ is the highest solar wave at noon, which is equal to 0.131 (m Q); Q represents the total daily radiation of the sun in units of J/m^2^; *c* represents the effective total daily solar radiation in hours; *m* is the coefficient of solar distribution, which is equal to 12/*c*; and *ω* stands for angular frequency (*ω* = 2Π/24 (rad). The heat transfer happens between both the asphalt road surface and the nearby air during the thermal conductivity. The convection heat transfer ($q_{c}$) can be measured based on the Newton convection/cooling equation, which is given as:

(10) $$q_{c}=\beta \cdot A\cdot \left(T-T_{a}\right)$$

Here, *β* is the coefficient of convection heat movement in unit W.m^−2^. K^−1^, which is equal to *β* = 3.0 υ + 5.6; υ stands for wind speed measured in unit m/s; *A* stands for contact area in m^2^; and *T_a_* stands for air temperature measured in °C. While emitting radiation from the environment, the asphalt also emits heat energy from long-wave radiation. Equation (11) can be used to measure the thermal irradiation ($q_{r}$) among the road surface and bordering air according to the Stefan–Boltzmann law.

(11) $$q_{r}=C_{s}\times e\times \left[\left(T-T^{*}\right)-\left(T_{a}-T^{*}\right)\right]$$

Here, *e* is the coefficient of emissivity of the road surface (*e* = 0.81); *C* is the Stefan–Boltzmann constant $C_{s}=5.6697\times 10^{-8}{\;\mathrm{Wm}}^{-2}\cdot K^{-4}.$ T* is used for the absolute temperature at zero degrees and it is equal to −273 °C.

By combining Equations (9)–(11), the temperature of the road surface under the boundary conditions could be determined with the help of Equation (12).

(12) $$\lambda \left(\frac{\partial T}{\partial x}n_{x}+\frac{\partial T}{\partial y}n_{y}+\frac{\partial T}{\partial z}n_{z}\right)=q_{s}+\beta \cdot A\cdot \left(T-T_{a}\right)+C_{s}\times e\times \left[\left(T-T^{*}\right)-\left(T_{a}-T^{*}\right)\right]$$

## 5. Conclusions

In this study, two waste polymer plastics, PET and PE, are employed as a bitumen modifier to optimize the structural and engineering properties of the asphalt mixtures using RSM based on Box–Behnken design (BBD). The effects of three parameters on the Marshall characteristics were studied and analyzed in this study. The key findings of the present study are concluded as:

- The research suggests that employing domestic polymer waste, namely PET and PE plastic, as a modifier in 60/70 grade bitumen and pavement mixtures is a promising and novel strategy.
- The current study investigated three parameters of Marshall characteristics for pure and polymer-modified asphalt binder and mixtures. There is only one parameter, MF, which is not optimized to within the standard range by RSM. Statistical analyses could not be performed for the MF response owing to a lack of data point diversity. This could be due to too large runs of data or range.
- The optimization of responses is determined by RSM is as follows: MS is 42.98 kN, MF is 5.08 mm, and MQ (kN/mm) is 8.66, indicating favorable and consistent precision when compared with experimental values.
- The incorporation of both waste polymers (PET and PE) dramatically reduces penetration (0.1 mm) and ductility (cm) measurements while increasing flashpoint (°C) and softening point (°C) readings compared with the corresponding standard range.
- The polymer-modified mixtures had better stability strength than the control specimen, allowing them to withstand heavy traffic loads and local environmental conditions.
- Similarly, the increased Marshall quotient (stability/flow) readings showed that asphalt mixtures modified with both polymer materials became stiffer and showed great resilience against permanent deformation after 1 and 30 days of testing when placed in local environmental conditions.
- The use of polymer waste contents in asphalt mixtures during road construction will help to extend the expected lifespan of our roadways and assist in the diversion of millions of metric tons (MMT) of plastic wastes from landfills; both of these are positive outcomes.

## 6. Recommendations and Limitations

- Proactive rules, monitoring, and assistance are needed, particularly in developing countries, to encourage the utilization of polymer waste (i.e., PET and PE) by road companies and organizations in the construction and maintenance of road projects.
- Municipal waste management organizations should also establish appropriate waste disposal mechanisms to allow for the distinct collection and disposal of plastic, in addition to addressing this major current issue.
- Future testing should include a variety of mixing circumstances to determine the strength and fatigue properties of PET- or PE-modified asphalt mixes. Moreover, future research could also include a comparison of PET concentration variations over time, as well as aging scenarios paired with UV exposure.
- In addition, the effect of temperature variation on the road surface due to environmental conditions with new parameters is also recommended and will be studied in the future for a better understanding of the behaviour of asphalt modified with plastic wastes.

## Figures and Tables

**Table 1:** Physical properties of polymer-modified binders.

**Test**	**Units**	**Results**	**Limits**	**Test Standard**	**Remarks**
Bitumen (Binder)					
Ductility @ 25 °C	cm	95	>75	ASTM D-113	ok
Penetration @ 25 °C	mm	64	60–70	ASTM D-5	
Flash Point	°C	302	232 min	ASTM D-92	
Softening Point	°C	55	40–55	ASTM D-36	
Aggregates					
Water Absorption	%	1.87	<2	ASTM C-127	ok
Specific Gravity	-	2.27	2–3		
Aggregate Crushing	%	24	<30	BS 812-3	
Aggregate Impact Value	%	19	<27		
Los Angeles Abrasion	%	31	<35	ASTM C-131	
Bulk Density	kg/m^3^	1500	-	ASTM C-29	
Elongation Index	%	29.44	<45	BS 812-105.2	
Flakiness Index	%	21	<30	BS 812-105.1	

**Table 2:** Experimental design for Box–Behnken design (BBD)-based RSM.

Factors	Units	Experimental Levels in BBD-Based RSM	
		Low Level (−1)	High Level (+1)
A: Polymer Type	-	0	1
B: Polymer Content	%	0	20
C: Testing Days	Day	1	30

**Table 3:** Experimental design of polymer-modified asphalt mixtures using RSM.

No.	Factors (Independent)			Response Variables		
	X1: PT	X2: PC (%)	X3: Day	Y1: MS (kN)	Y2: MF (mm)	Y3: MS (kN/mm)
1	1	0	30	29.21	7.1	4.11
2	0	0	1	15.56	3.78	4.11
3	0	20	1	10.06	2.1	4.79
4	0	0	30	29.21	7.1	4.11
5	0	20	30	14.09	5.34	2.63
6	1	20	30	43.91	5.07	8.66
7	1	0	1	15.56	3.78	4.11
8	1	20	1	30.03	2.02	14.86
9	1	20	30	43.91	5.07	8.66
10	1	0	30	29.21	7.1	4.11
11	0	0	1	15.56	3.78	4.11
12	0	0	30	29.21	7.1	4.11
13	0	20	30	14.09	5.34	2.63
14	1	20	1	30.03	2.02	14.86
15	0	20	1	10.06	2.1	4.79
16	1	0	1	15.56	3.78	4.11
17	0	20	30	14.09	5.34	2.63
18	0	20	1	10.06	2.1	4.79
19	1	20	30	43.91	5.07	8.66
20	1	20	1	30.03	2.02	14.86
21	1	0	1	15.56	3.78	4.11
22	0	0	30	29.21	7.1	4.11
23	1	0	30	29.21	7.1	4.11
24	0	0	1	15.56	3.78	4.11

**Table 4:** ANOVA results for responses and factors of modified asphalt mixture.

Responses-Y	Factors	Sum of Sq.	dF	Mean Sq.	F-Value	*p*-Value	Significant
Y1: MS (kN)	Model	28.93	6	4.82	605.04	<0.0001	Yes
	A: Polymer Type	10.07	1	10.07	1263.17	<0.0001	*
	B: Polymer Content	0.0417	1	0.0417	5.23	0.0372	*
	C: Testing Days	8.10	1	8.10	1016.83	<0.0001	*
	AB	10.07	1	10.07	1263.17	<0.0001	*
	AC	0.1195	1	0.1195	15.00	0.0015	**
	BC	0.5325	1	0.5325	66.82	<0.0001	*
	Residual	0.1195	15	0.0080			
Y2: MF (mm)	Model	4.90	6	0.8174	33,359.91	<0.0001	Yes
	A: Polymer Type	0.0028	1	0.0028	115.96	<0.0001	*
	B: Polymer Content	1.19	1	1.19	48,744.70	<0.0001	*
	C: Testing Days	3.68	1	3.68	1.502 × 10^5^	<0.0001	*
	AB	0.0028	1	0.0028	115.96	<0.0001	*
	AC	0.0004	1	0.0004	15.00	0.0015	**
	BC	0.0237	1	0.0237	967.11	<0.0001	*
	Residual	0.0004	15	0.0000			
Y3: MQ (kN/mm)	Model	337.86	7	48.27	8.500 × 10^11^	<0.0001	Yes
	A: Polymer Type	97.18	1	97.18	1.711 × 10^12^	<0.0001	*
	B: Polymer Content	78.79	1	78.79	1.388 × 10^12^	<0.0001	*
	C: Testing Days	26.22	1	26.22	4.618 × 10^11^	<0.0001	*
	AB	97.18	1	97.18	1.711 × 10^12^	<0.0001	*
	AC	6.16	1	6.16	1.085 × 10^11^	<0.0001	*
	BC	26.16	1	26.16	4.608 × 10^11^	<0.0001	*
	ABC	6.16	1	6.16	1.085 × 10^11^	<0.0001	*
	Residual	7.950 × 10^−10^	14	5.678 × 10^−11^			

Note: “*” *p* < 0.0001; “**” *p* > 0.0001.

**Table 5:** Optimal prepared factors values in BBD-based RSM (predicted) and experimentally.

Y: Responses	Optimal Factors in BBD-Based RSM			Optimal Y-Values	
	X1: PT	X2: PC (%)	X3: Testing Day	Predicted	Experimental
Y1: MS (kN)	0.98	20	30	42.98	43.84 ± 0.30
Y2: MF (mm)	1	20	30	5.08	4.61 ± 0.18
Y3: MQ (kN/mm)	1	20	29.5	8.75	8.68 ± 0.12

## Data Availability

Data will be available on suitable demand.
